# Genomic Tracing Reveals Multiple Independent Occurrences of *Bactrocera dorsalis* in Belgium

**DOI:** 10.3390/insects16121271

**Published:** 2025-12-15

**Authors:** Sam Vanbergen, Pablo Deschepper, Jan Van Autreve, Vera Huyshauwer, Massimiliano Virgilio, Jochem Bonte, Wannes Dermauw

**Affiliations:** 1 Biology Department, Royal Museum for Central Africa (RMCA), Leuvensesteenweg 13, 3080 Tervuren, Belgium; massimiliano.virgilio@africamuseum.be; 2 Ecology, Evolution and Conservation Biology, Biology Department, KU Leuven, 3000 Leuven, Belgium; 3 Biology Department, Royal Belgian Institute for Natural Sciences (RBINS), Vautierstreet 29, 1000 Brussels, Belgium; pdeschepper@naturalsciences.be; 4 Federal Agency for the Safety of the Food Chain (FASFC), Kruidtuinlaan 55, 1000 Brussel, Belgium; 5 Plant Sciences Unit, Flanders Research Institute for Agriculture, Fisheries and Food (ILVO), Burgemeester Van Gansberghelaan 96, 9820 Merelbeke-Melle, Belgium

**Keywords:** oriental fruit fly, incursion, geographic origin, SNP, whole-genome sequencing, DAPC, COI barcode, mitonuclear discordance

## Abstract

*Bactrocera dorsalis* (Hendel) is a major insect pest of commercial fruit and a quarantine priority in the European Union (EU). This tephritid species was previously recorded in Austria, France, and Italy, with more recent detections in Greece. In 2023 and 2024, *B. dorsalis* adult males were captured by the Federal Agency for the Safety of the Food Chain (FASFC) using traps placed in community gardens and produce markets in Belgium. Morphological identification confirmed the specimens as *B. dorsalis*. Genomes of trapped adults were sequenced, as well as a historical set of *B. dorsalis* larvae intercepted by FASFC from imported fruit. A nuclear single-nucleotide polymorphism (SNP) analysis revealed that three Belgian *B. dorsalis* adults originated from Africa, while eight others were of Asian origin. In contrast, almost all FASFC intercepted larvae had an African origin. A discriminant analysis of principal components (DAPC) of the “Folmer” region of the mitochondrial cytochrome c oxidase subunit I (COI) gene largely confirmed the SNP analysis but also indicated that, in a few cases, mitonuclear discordance may confound origin tracing. To enable geographic origin tracing in a laboratory setting, a diagnostic set of nuclear SNPs was developed. The DAPC was also implemented in a streamlined R-script, allowing origin assignment using a mitochondrial COI barcode. To conclude, our study reveals independent occurrences of *B. dorsalis* in Belgium and provides important tools for origin tracing of this quarantine pest.

## Simple Summary

The oriental fruit fly (*Bactrocera dorsalis*) is a destructive fruit pest of quarantine concern in the European Union (EU). Until recently, it had not been reported in more northern regions of the EU. In 2023 and 2024, adult males were captured in Belgium using monitoring traps in community gardens and produce markets. Genome-wide analyses of nuclear DNA showed that some flies originated from Africa, while others were of Asian origin. Mitochondrial DNA fragment analysis produced similar results, though with lower accuracy in some cases. To facilitate efficient geographic origin tracing of individual *B. dorsalis* specimens, a genetic approach was developed based on two nuclear DNA markers. In addition, an easy-to-use R script was developed to trace *B. dorsalis*’ origin via a mitochondrial DNA fragment. Our results demonstrate multiple independent introductions of *B. dorsalis* into Belgium and provide molecular tools for origin tracing of *B. dorsalis.*

## 1. Introduction

True fruit flies (Diptera: Tephritidae) are a diverse group of insects, comprising nearly 5000 species distributed across more than 500 genera worldwide [1,2]. These flies predominantly thrive in (sub)tropical environments, where they play various ecological roles. Several species within this family are notorious for their economic impact, causing billions of euros in crop losses annually [3]. Many tephritid pests, particularly those in the *Bactrocera* genus, infest a wide variety of commercial fruit and vegetables. Among them, the oriental fruit fly, *Bactrocera dorsalis* (Hendel), is the most infamous, being widely recognized as a highly invasive and destructive pest. Native to Asia, it has now spread to more than 60 countries, including those in Oceania and sub-Saharan Africa [4]. For the latter region, *B. dorsalis* was first reported in East African countries before rapidly spreading to central, West and southern Africa [5].

*Bactrocera dorsalis* is part of a group of closely related species known as the *B. dorsalis* complex, which includes over 75 species [6,7,8]. Identifying specimens within this complex is challenging due to morphological similarities and overlapping characteristics [9,10]. The taxonomy of species in the *B. dorsalis* complex is uncertain, and hybridization within the complex has been reported, further complicating accurate identification [8,11,12]. However, by combining mitochondrial and nuclear markers, *B. dorsalis* can be distinguished from morphologically similar species and other closely related taxa [13].

In the European Union (EU), *B. dorsalis* is listed as a quarantine pest in Implementing Regulation (EU) 2019/2072 (Annex II A), meaning its introduction into the EU is prohibited due to the risk it poses to EU crops [14]. Furthermore, Delegated Regulation (EU) 2019/1702 [15] designates *B. dorsalis*, along with three other tephritid species, as a priority pest because of its potential to cause the most severe economic, environmental, and social impact. In line with this, the European Food Safety Authority (EFSA) estimates that *B. dorsalis* could cause average yield losses of around 10% in citrus and stone fruit crops within the EU [16].

Eggs and larvae of *B. dorsalis* are frequently intercepted at EU points of entry, as recorded in the EUROPHYT database [17]. In Belgium, for example, the majority of *B. dorsalis* eggs and larvae are detected in mangoes imported from West African countries [18]. In recent years, occurrences of *B. dorsalis* adults have been reported in several EU countries, including Austria (with the longest history of detection), France, Italy, and Greece, but not in countries located further north [19,20,21,22,23,24,25].

In the summer of 2023, the Belgian National Plant Protection Organization (NPPO), more specifically the Federal Agency for the Safety of the Food Chain (FASFC), detected *B. dorsalis* for the first time in Belgium. The initial specimen was recovered from a methyl eugenol-baited trap during routine monitoring in Antwerp city, with additional captures reported in two other cities [26]. These detections triggered an expanded survey to assess the pest’s presence and potential spread. We initiated the current study to determine the geographic origin of the trapped specimens and assess whether they resulted from a single introduction or multiple incursions—critical information for identifying introduction pathways and guiding phytosanitary measures at national and EU levels.

To address this aim, whole-genome sequencing (WGS) was performed on DNA extracted from trapped adults and historical interceptions of larva. Both nuclear as well as mitochondrial DNA analyses were performed to infer the geographic origin of the *B. dorsalis* specimens. Additionally, the feasibility of developing a reduced nuclear single-nucleotide polymorphism (SNP) panel to distinguish broad origin regions—Africa versus Asia—was assessed. Such a panel, compatible with probe-based qPCR or KASP assays, could provide a rapid, cost-effective tool for tracing future *B. dorsalis* incursions.

## 2. Materials and Methods

### 2.1. Survey and Sample Collection

Surveys for *B. dorsalis* adults were conducted by FASFC in Belgium during 2023 and 2024 using methyl eugenol-baited plastic McPhail type traps. These surveys were performed annually between June and October at various locations, including points of entry (Antwerp, Merksem, Kallo, Beveren-Waas, Meer, Machelen, Grâce-Hollogne), fruit and vegetable wholesalers (Brussels, Asse, Hoogstraten, Liège, Ans, Charleroi, Mons, Beveren-Kruibeke-Zwijndrecht), and produce markets (Anderlecht, Courcelles, Lokeren, Herstal, Antwerp). In early August 2023, the first *B. dorsalis* adult male (Bd_2023_1) was detected in a trap at the public produce market “Theaterplein” in Antwerp. A few weeks later, two more adult males (Bd_2023_2 and Bd_2023_3) were caught at public produce markets in Anderlecht (“Abbatoir”) and Courcelles (“Place Roosevelt”), approximately 50 km and 100 km from the initial detection site, respectively [26]. In response, FASFC added additional public spaces in Antwerp—including street parks and community gardens—to their survey to assess the presence and potential spread of *B. dorsalis*. Between September and October 2023, four additional adult males (Bd_2023_4–7) were found in traps at community gardens (e.g., “De Verborgen Kloostertuin”, “Samentuin Lijsterbes”) and small street parks (e.g., “Maurits Sabbelaan”, “Serviceflat Kerkeveld”) in Antwerp.

**Figure 1:**
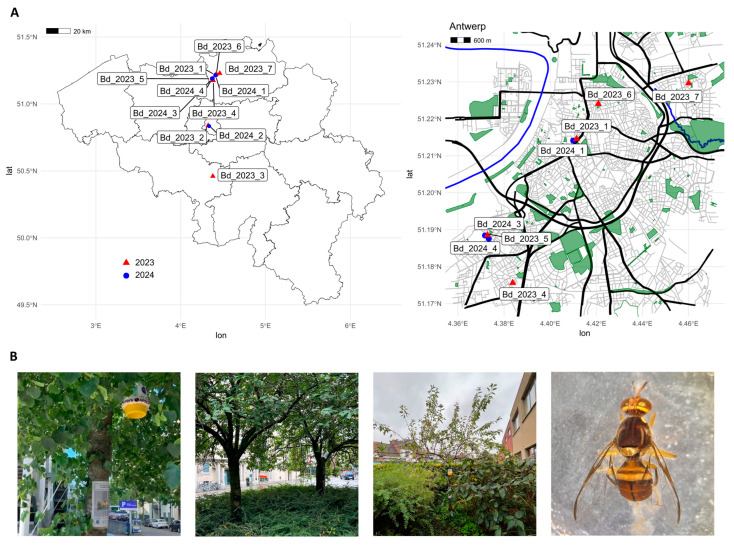
Locations of *Bactrocera dorsalis* adult males trapped in Belgium during 2023–2024. ((**A**)**-left**) Map of Belgium, with location of the eleven *Bactrocera dorsalis* adult males that were trapped in 2023 (red triangle) and 2024 (blue circle); ((**A**)**-right**) Map of Antwerp showing the locations of the five and three *Bactrocera dorsalis* adult males that were trapped in 2023 (red triangle) and 2024 (blue circles), respectively. (**B**) trap at “Theaterplein” in Antwerp (Bd_2023_1), trap in a *Prunus* tree at “Maurits Sabbelaan” in Antwerp (Bd_2023_5, Bd_2024_3, Bd_2024_4), trap at ‘serviceflat Kerkeveld’ in Antwerp (Bd_2023_7) and a *Bactrocera dorsalis* adult male found in the “Maurits Sabbelaan’ in 2024 (Bd_2024_4).

In 2024, the same locations were resurveyed by FASFC, with the addition of two wholesalers in Sint-Katelijne-Waver and Tournai, two produce markets in Bruges and Eghezée, and composting facilities in Brussels, Merksplas, Brecht, Herstal, Fleurus, Quévy, and Beveren-Kruibeke-Zwijndrecht. In Antwerp, only those public spaces where *B. dorsalis* had previously been trapped were included. That year, three more adults (Bd_2024_1, Bd_2024_3, Bd_2024_4) were captured in Antwerp at previously positive sites (“Theaterplein” and “Maurits Sabbelaan”), and again one adult (Bd_2024_2) was found at the public market in Anderlecht (Figure 1, Table 1, Supplementary Table S1).

Morphological identification of trapped adult specimens suspected to be *B. dorsalis* was performed by the National Reference Laboratory (NRL) at the Flanders Research Institute for Agriculture, Fisheries and Food (ILVO) and the Royal Museum for Central Africa (RMCA). Identified specimens (Bd_202#_#) were preserved in 1.5 mL Eppendorf tubes containing 100% ethanol, and stored at 4 °C until DNA extraction. In addition to surveys, FASFC intercepted immature *B. dorsalis* specimens (larvae) during inspections of imported fruits, primarily mangoes. Larvae were identified by the NRL of ILVO as *B. dorsalis* and preserved in 100% ethanol at 4 °C, stored at −80 °C, or in plant lysis buffer (EXT-001, Optigene, Horsham, United Kingdom) at −20 °C until DNA extraction (“FAVV_” samples). These larvae predominantly originated from West African countries, including Senegal (*n* = 4), Ivory Coast (*n* = 3), Cameroon (*n* = 2), and Burkina Faso (*n* = 1), with one additional sample from Bangladesh (Table 1).

To provide a broader context for the genetic analyses, individuals from a *B. dorsalis* population collected in Saint Paul, Réunion, in July 2023 (“GSP” samples, collected during the REACT project) were included, preserved in 100% ethanol and stored at −20 °C until DNA extraction. Importantly, raw sequencing data from Zhang et al. [27] were also downloaded from CNGBdb (https://db.cngb.org/search/project/CNP0002783/; accessed on 15 December 2023), and in this study referred to as the “WGS reference dataset.” This dataset comprises 487 individuals from 50 *B. dorsalis* populations and 25 *B. carambolae* individuals from three populations, serving as reference samples. Of particular note, sequencing data from two *B. dorsalis* individuals of the WGS reference dataset (MGAT07 and MGAT08) were not included in our analyses as the sequencing files of these individuals were corrupted. Metadata at both population and individual levels are provided in Supplementary Tables S2 and S3.

**Table 1:** FASFC *Bactrocera dorsalis* samples—adult males found in traps in Belgium (Bd_202#_# samples ^a^) or larvae intercepted from imported mangoes (FAVV_# samples ^b^)—used in this study. For more detailed info see Supplementary Table S1.

FASFC ID	Date Found	Sample Name	Trap/Host	Country	Latitude	Longitude	Storage Method (Until DNA Extraction)
2926-23-0035	4 August 2023	Bd_2023_1 ^a^	Trap	Belgium	51.214793	4.410569	100% EtOH, 4 °C
3453-23-0065	29 August 2023	Bd_2023_2 ^a^	Trap	Belgium	50.842950	4.327830	100% EtOH, 4 °C
3450-23-0067	24 August 2023	Bd_2023_3 ^a^	Trap	Belgium	50.460712	4.378491	100% EtOH, 4°C
2302-23-0031	8 September 2023	Bd_2023_4 ^a^	Trap	Belgium	51.175888	4.383611	100% EtOH, 4 °C
2302-23-0044	22 September 2023	Bd_2023_5 ^a^	Trap	Belgium	51.188346	4.372646	100% EtOH, 4 °C
2372-23-0077	25 September 2023	Bd_2023_6 ^a^	Trap	Belgium	51.223419	4.4205927	100% EtOH, 4 °C
2126-23-0053	9 October 2023	Bd_2023_7 ^a^	Trap	Belgium	51.229344	4.4602415	100% EtOH, 4 °C
2372-24-0181	25 July 2024	Bd_2024_1 ^a^	Trap	Belgium	51.214793	4.410549	100% EtOH, 4 °C
3453-24-0075	6 August 2024	Bd_2024_2 ^a^	Trap	Belgium	50.842950	4.327830	100% EtOH, 4 °C
2992-24-0040	23 August 2024	Bd_2024_3 ^a^	Trap	Belgium	51.188346	4.372646	100% EtOH, 4 °C
2257-24-0079	6 September 2024	Bd_2024_4 ^a^	Trap	Belgium	51.188346	4.372646	100% EtOH, 4 °C
4703-24-0048	19 March 2024	FAVV_1 ^b^	Mango	Cameroon	-	-	−80 °C
7042-24-0048	1 May 2024	FAVV_2 ^b^	Mango	Cameroon	-	-	−80 °C
2870-24-0029	21 May 2024	FAVV_3 ^b^	Mango	Ivory Coast	-	-	−80 °C
2799-24-0104	24 May 2024	FAVV_4 ^b^	Mango	Ivory Coast	-	-	−80 °C
4703-24-0116	25 July 2024	FAVV_5 ^b^	Mango	Senegal	-	-	−80 °C
4825-24-0020	6 May 2024	FAVV_6 ^b^	Mango	Ivory Coast	-	-	100% EtOH, 4 °C *
2855-24-0018	8 August 2024	FAVV_7 ^b^	Mango	Senegal	-	-	lysis buffer, −20 °C **
7022-22-0013	13 May 2022	FAVV_11 ^b^	Mango	Burkina Faso	-	-	100% EtOH, 4 °C
2598-23-0137	27 July 2023	FAVV_12 ^b^	Mango	Senegal	-	-	100% EtOH, 4 °C
2854-23-0082	3 August 2023	FAVV_13 ^b^	Mango	Senegal	-	-	100% EtOH, 4 °C
7075-21-0048	14 July 2021	FAVV_14 ^b^	Mango	Bangladesh	-	-	100% EtOH, 4 °C

* larva was boiled in distilled water before being stored in ethanol, ** the larva was stored in plant lysis buffer, as this buffer was used to generate the DNA extract for a LAMP assay [28] performed on this larva. After completing the LAMP assay, the buffer containing the larva was stored at −20 °C until a second DNA extraction was performed on the larva (without buffer) using the Qiagen Blood & Tissue DNA extraction kit (Qiagen, Antwerp, Belgium).

### 2.2. Genomic DNA Extraction

DNA extractions from three adult *B. dorsalis* males trapped in 2023 (Bd_2023_1, Bd_2023_2, Bd_2023_3) were performed at GenomeScan (Leiden, The Netherlands), using a MagNA Pure 96 kit (Roche, Woerden, The Netherlands). All other DNA extractions (either FASFC samples or samples from Réunion) were carried out at RMCA or ILVO using the Qiagen DNeasy Blood & Tissue Kit (Qiagen, Antwerp, Belgium), following the manufacturer’s protocol, with elution volumes ranging from 30 to 100 µL. DNA concentrations were assessed via spectrophotometric methods using either an Implen Photometer (Implen, Munich, Germany; at ILVO) or a Qubit 4 fluorometer (Thermo Fisher Scientific, Merelbeke-Melle, Belgium; at RMCA). All DNA samples were stored at −20 °C until sequencing.

### 2.3. Sequencing

DNA integrity was assessed using an Agilent fragment analyzer (Agilent Technologies, Diegem, Belgium). For three adult *B. dorsalis* specimens from 2023 (Bd_2023_1, Bd_2023_2 and Bd_2023_3), 2 × 150bp paired-end Illumina sequencing of genomic DNA was performed at GenomeScan (Leiden, The Netherlands) using a NovaSeq 6000 device. Sequencing of genomic DNA from the remaining *B. dorsalis* adults trapped in 2023, the *B. dorsalis* adults trapped in 2024, the intercepted larvae, and individuals from Réunion was performed at Novogene (Munich, Germany). At Novogene, DNA libraries (with a 350 bp insert) were created and loaded on a NovaSeq X plus device for 2 × 150 paired-end Illumina sequencing.

### 2.4. Read Mapping, Quality Control and Variant Calling

Raw sequencing reads were filtered and trimmed using fastp v0.23.4 with the following options: “—qualified_quality_phred 20—correction—length_required 75” [29]. For quality control of the newly sequenced individuals fastp reports were combined into a single QC report with multiqc v1.25.1 [30]. Next minimap2 v2.26 was used to map the trimmed and filtered reads to the *B. dorsalis* NCBI RefSeq genome assembly GCF_023373825.1 with the short read mapping option (-ax sr) [31]. The resulting BAM files were sorted and marked for duplicates with samtools v1.20 using the sort, fixmate and markdup subcommands [32]. To assess the quality of each BAM file, depth of coverage was calculated using mosdepth v0.3.11 [33], and a summary of mapping statistics was generated with samtools flagstat [32]. To standardize sequencing depth across samples, BAM files with excessively high read depth were downsampled using samtools sample. The sampling fraction was calculated as the ratio of the target median depth to the original median depth of each sample [32].Variants were then called with ANGSD v0.940 [34], retaining only properly paired reads with mapping quality > 30 and base quality > 30. Sites with depth <2 and >15 were discarded as well. In addition to the individual-level filters mentioned above, only sites with 2160 < total depth < 8100 were retained, corresponding to 4× and 15× times the number of individuals in the cohort as lower and upper depth limit, respectively. Finally, loci with missing data in more than 10% of the individuals and minor allele frequency below 0.05 were discarded. Genotype likelihoods (GLs) were exported in Beagle format, and both GLs and called genotypes (GTs) were provided in VCF format. Genozip v14.0.35 was used for lossless fastq and bam file compression and decompression to manage disc space in between steps of the analysis [35].

### 2.5. PCA of Inferred Nuclear Allele Frequencies

Genetic relatedness among samples was assessed using nuclear SNP allele-frequency data. Genotype likelihoods (GLs) from the variant calling analysis were used as input for PCAngsd v1.21 to estimate a sample-by-sample covariance matrix. This was performed by iteratively inferring allele frequencies from the GLs [36]. This approach incorporates the uncertainty of genotypes (GTs) in the form of GLs within the analysis without the need for strict SNP and/or individual GT filtering prior the analysis, facilitating the use of low depth and/or coverage samples. Sample relationships were visualized using principal component analysis (PCA) and neighbour-joining (NJ) tree, as implemented in PCAngsd, based on the inferred covariance matrix.

### 2.6. Identification of Diagnostic SNP Set

**Figure 2:**
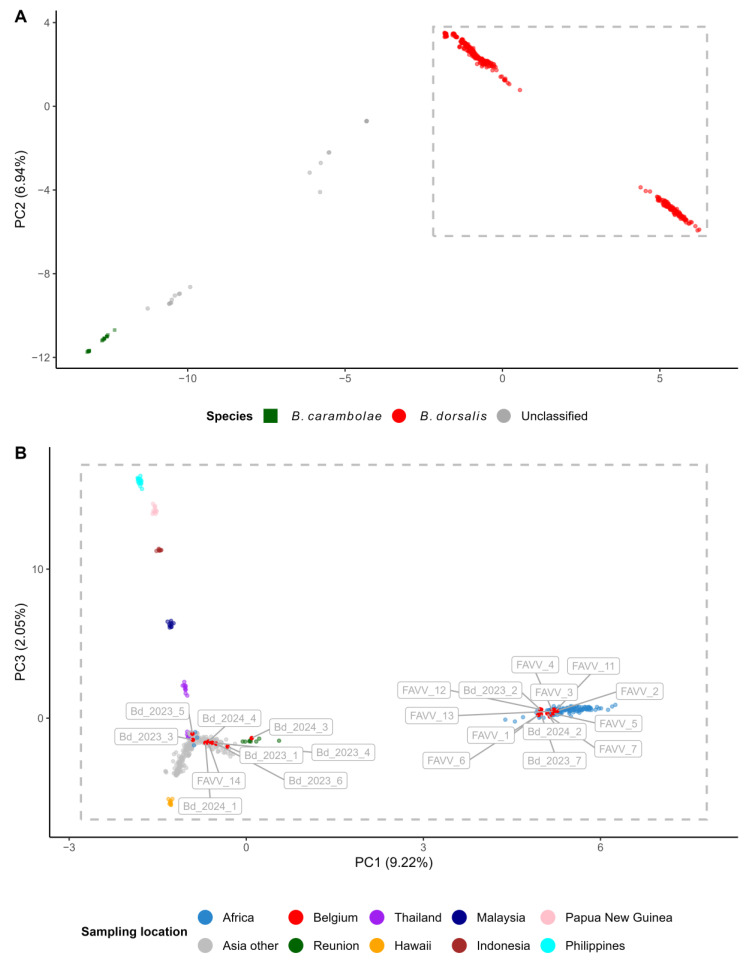
Principal component analyses (PCA) of nuclear allele frequencies. (**A**) PCA of nuclear SNP frequencies identified in *Bactrocera dorsalis* and *Bactrocera carambolae* individuals (PC1 vs. PC2). A large group of *Bactrocera dorsalis* individuals (462 from the WGS reference dataset, 9 from Réunion, and 22 trapped/intercepted by FASFC) is indicated with red dots (framed with a dashed rectangle), while 22 PCA outliers and 25 *Bactrocera carambolae* specimens are indicated with grey dots and dark green squares, respectively. (**B**) Detail of the large group of *Bactrocera dorsalis* individuals (red dots, grey rectangle) in panel (**A**). The adults (Bd_202#_#) and larvae (FAVV_#) that were trapped or intercepted in Belgium, respectively, are indicated with a red dot and have a framed label. Three blue dots on the left side of the PCA plot are individuals from South Africa (ZAMP06, ZAMP07 and ZAMP09) that did not cluster with the other African *Bactrocera dorsalis* samples (on the right side of PCA plot).

A subset of *B. dorsalis* individuals—comprising all nine specimens from Réunion, along with those from the WGS reference that clustered within either the African or Asian group in the PCA (462 individuals, Figure 2)—was used to identify multiple SNP panels for tracing the origins of trapped or intercepted specimens. These SNP panels were identified using a machine learning approach with the ‘rfe’ (recursive feature elimination) function from the ‘caret’ package v7.0-1 in R using a random forest model [37]. First, the subsetted vcf file was filtered with bcftools to retain genomic sites with no more than 1% missing data, minor allele frequency above 5% and an average depth between 8 and 12. The remaining missing genotypes were imputed with the ‘rfImpute’ function of the ‘randomForest’ package [38]. The genotype matrix was split into a training dataset and an out-of-bag (30%) set for internal validation of classification accuracy. The classification accuracy was assessed for SNP panels of various sizes (1, 2, 5, 10, 15, 20, 50, 80, 100, 150 and 200 SNPs) using 10-fold cross-validation and repeated 10 times. After identifying the best-performing diagnostic SNP set, a new random forest model was trained using only the selected SNPs, with 25% of the data withheld for validation through the ‘predict’ function from the ‘stats’ package in R. This validation process was repeated 100 times, each with a different validation subset [39]. Finally, to further reduce the number of predictor loci while maintaining discriminatory power, a decision tree was built using the ‘rpart’ function v4.1.24 in R, based on the SNPs selected through recursive feature elimination [40]. This decision tree was applied only to trapped/intercepted individuals with complete data for the retained diagnostic SNPs. Individuals with loci sequenced at a depth below six were conservatively excluded, as such loci were treated as missing to minimize the risk of allelic dropout at heterozygous sites.

### 2.7. DAPC of COI DNA Barcodes

To independently validate the nuclear SNP-based origin tracing, a mitochondrial haplotype-based origin tracing was employed using the cytochrome c oxidase subunit I (COI) 5’ gene fragment—commonly referred to as the “COI DNA barcode” or “Folmer” region of the COI gene (see Doorenweerd et al. and references therein) [41]. WGS data were first used to reconstruct complete mitochondrial genomes (mitogenomes) for all individuals. Reads of trapped flies/intercepted larvae and those in the WGS reference dataset, were mapped to a *B. dorsalis* mitochondrial reference genome (Genbank accession NC_008748.1). Reads were quality-filtered before mapping using fastp, applying a base quality threshold of Phred 30 (--qualified_quality_phred 30). After mapping, a mitochondrial haplotype was achieved with samtools consensus (v1.20) with option “--mode Bayesian --cutoff 30 --format FASTA --min-MQ 40 --P-het 0”. Additionally, *B. dorsalis* COI DNA barcodes were downloaded from the BOLD Systems database (www.boldsystems.org, accessed on 15 January 2025; see Supplementary Table S4). The reconstructed mitogenomes were aligned with the downloaded COI sequences using the FFT-NS-2 algorithm of MAFFT v7.526 [42]. An alignment of the 658 bp “Folmer” region was then excised from the total alignment. The alignment was manually curated through multiple filtering steps to reduce alignment artefacts, including stop codons, inconsistent gaps, and putative sequencing errors. This process included visual inspection and manual removal of problematic sequences using Jalview (v2.11.4.1) [43]. Using the R package adegenet (v2.1.11) [44], the final alignment was converted into a genotype matrix (function DNAbin2genind) for population genetic analyses.

The ‘find.clusters’ function was used to infer clusters of related COI haplotypes. Successive k-means clustering was performed for an increasing number (k) of clusters, after summarizing the COI variation with PCA. Specifically, the 80 most important principal components (PCs), capturing >90% of total variation in the dataset, were initially retained. Clustering was explored for values of k from 1 to 40, and k = 10 was selected based on the “diffNgroup” criterion as a suitable number of clusters (Supplementary Figure S1). Assignment performance was evaluated using the ‘xvalDapc’ function to identify the optimal number of PCs. The full dataset was randomly split into 90% training and 10% test sets, repeated over 30 iterations. The highest mean assignment accuracy (97.7%) was achieved with 25 PCs. Accordingly, the final Discriminant Analysis of Principal Component (DAPC) model was fitted on the 25 first PCs using nine (K–1) discriminant axes, and the cluster groupings defined with the ‘find.clusters’ step. Within each DAPC cluster, normalized proportions of individuals from different geographic areas were calculated to account for unequal sampling effort. *B. dorsalis* accessions lacking recorded sampling locations were excluded from the calculation of regional proportions. Accessions originating from regions without confirmed established populations, as well as those from areas with insufficiently documented records (e.g., West Asia), were also omitted from this analysis.

### 2.8. Whole-Mitogenome Phylogeny

Given the known challenges in the morphological and molecular systematics of the *B. dorsalis* complex, such as mitochondrial introgression, incomplete lineage sorting, hybridization, unstable taxonomy, which could potentially affect molecular identification [12,45,46,47,48,49], a phylogenetic analysis of mitochondrial genomes was performed to better support the interpretation of origin tracing via DAPC of COI barcodes. With this purpose, 21 complete mitogenomes of *Bactrocera* spp. were downloaded from NCBI GenBank (see Supplementary Table S5) and aligned with the reconstructed mitogenomes of the 540 individuals (509 from the WGS reference dataset, nine Réunion “GSP” individuals, 22 trapped/intercepted specimens) described in Section 2.7. After alignment with the MAFFT FFT-NS-2 algorithm, manual curation was performed in Jalview as described above [42,43]. The resulting alignment was used for maximum-likelihood tree reconstruction with FastTree v2.2 [50] with default settings (using a Jukes-Cantor nt substitution model). To assess node support, 200 bootstrap replicates were generated using the seqboot command from Phylip v3.697-2 [51]. Bootstrap values were then mapped onto the original tree using the CompareToBootstrap.pl script from FastTree and the tree visualized in R with ggtree [52]. To aid interpretation of clustering patterns, COI DNA barcode fragments extracted from mitogenomes used in the ML reconstruction were queried against the reference sequence library of BOLD Systems (accessed on 20 June 2025). An arbitrary threshold of 1% divergence was applied to the best close matches to better account for ambiguities arising from overlapping intra- and interspecific variation within the *B. dorsalis* complex.

## 3. Results

### 3.1. DNA Extraction, Sequencing, Read Mapping and Variant Calling

DNA quality checks, performed via spectrophotometry and capillary electrophoresis, showed that a substantial proportion of samples had DNA concentrations greater than 2 ng/µL (Supplementary Table S1). However, DNA extractions from the first three adult *B. dorsalis* specimens trapped in 2023 (Bd_2023_1, Bd_2023_2, Bd_2023_3) yielded markedly lower DNA concentrations compared to other samples. It remains unclear whether this was due to the extraction protocol used by the sequencing provider or the suboptimal condition of these particular specimens. To rule out the former, all subsequent DNA extractions were carried out by either RMCA or ILVO.

Several DNA samples were flagged by the sequencing facilities as fragmented. This likely resulted from prolonged exposure of adult *B. dorsalis* to trapping liquid, potentially for several weeks, and long-term storage of larval specimens in ethanol. Nonetheless, as a general strategy, all DNA samples were subjected to WGS, as this has proven to be a pragmatic and effective approach for short-read sequencing of degraded or low-concentration DNA [53].

The proportion of sequencing reads with a Phred quality score above Q30 was consistently high across all newly sequenced samples (90.8–96.5%), indicating robust sequencing accuracy (Supplementary Table S6). The mean yield of >Q30 reads was 17.28 Gb (+/− 5.1 SD) per sample. With the exception of Bd_2023_1, Bd_2023_2, and Bd_2023_3, duplication rates were low and GC content fell within the expected range of 37–40%. In contrast, Bd_2023_1, Bd_2023_2, and Bd_2023_3 exhibited markedly reduced sequencing performance. These samples displayed elevated GC content (57.3%, 47.4% and 51.2%, respectively), whereas Bd_2023_2 and Bd_2023_3 showed unusually high duplication rates (57.7% and 56.7%, respectively). Furthermore, all three samples yielded substantially lower sequencing output, with mean depths of 0.85×, 2.17×, and 1.12×, and median depths of 0×, consistent with poor coverage and indicative of low-quality input DNA.

To standardize coverage across samples, BAM files with a mean depth exceeding 14× were downsampled to 10× using the samtools view command (Supplementary Tables S7 and S8). The samtools flagstat results for both original and downsampled BAM files are provided in Supplementary Tables S7 and S8. ANGSD was used over traditional genotype calling methods due to the presence of three samples (Bd_2023_1 to Bd_2023_3) in the dataset that exhibited low sequencing depth (Supplementary Table S7). ANGSD is specifically designed to handle such cases [34] by working directly with GLs rather than relying on hard genotype calls, which can be unreliable when coverage is limited. After quality filtering, 3,036,714 biallelic SNPs were retained in the final dataset. All newly generated sequencing data have been deposited in the Sequence Read Archive (SRA) under BioProject accession number PRJNA1254113.

### 3.2. PCA of Inferred Nuclear Allele Frequencies for Origin Tracing

The PCA revealed a main group of 493 *B. dorsalis* individuals (462 individuals from the WGS reference dataset, nine from Réunion and 22 *B. dorsalis* specimens trapped/intercepted in Belgium; Figure 2A, red dots) that were clearly separated from the 25 *B. carambolae* individuals (Figure 2A, dark green squares). Remarkably, 22 *B. dorsalis* individuals from the WGS reference dataset did not cluster with the main group of *B. dorsalis* individuals and are hereafter referred to as “PCA outliers” (Figure 2A, grey dots). Nine of these PCA outliers were already discussed in Zhang et al. as hybrids [27], but the full list is provided in Supplementary Table S9. Based on the observed patterns, these outliers may have resulted from genuine evolutionary processes such as hybridization or incomplete lineage sorting [27], or alternatively, from specimen misidentification, mislabeling or contamination.

The first principal component explains 9.94% of all variation in the covariance matrix (Figure 2A, Supplementary Figure S2). After excluding individual BIBJ10 from Burundi due to its high proportion of missing data (96.7%; see Supplementary Table S10), PC1 clearly separated a main cluster comprising all African individuals—with the exception of three individuals from South Africa, ZAMP06, ZAMP07 and ZAMP09—from another large cluster of *B. dorsalis* individuals (Figure 2B). The latter cluster was resolved along both PC1 and PC2 into distinct groups corresponding to samples from the Philippines, Papua New Guinea, Indonesia, Malaysia, Thailand, Hawaii, and La Réunion. Samples from multiple Asian countries within this cluster also clustered together without forming distinct subgroups.

The *B. dorsalis* larvae intercepted by FASFC from fruit imports (FAVV_# samples), all of known geographic origin, clustered with reference specimens from their respective regions. This included FAVV_14, which originated from Asia, and all other FASFC samples of African origin. Among the trapped *B. dorsalis* adults, three individuals (Bd_2023_2, Bd_2023_7, and Bd_2024_4) grouped with African *B. dorsalis* reference specimens, whereas the remaining adults clustered with Asian specimens (Figure 2, Table 2, Supplementary Table S1). The NJ tree showed patterns consistent with the PCA results.

Remarkably, none of the individuals trapped at the same location—“Theaterplein” (Bd_2023_1 and Bd_2024_1) or “Maurits Sabbelaan” (Bd_2023_5, Bd_2024_4, and Bd_2024_5) in Antwerp, and “Abbatoir” (Bd_2023_2 and Bd_2024_2) in Anderlecht—clustered closely. Instead, two pairs of trapped individuals, Bd_2024_1/Bd_2024_4 (which clustered with Asian individuals in the PCA) and Bd_2023_7/Bd_2024_2 (which clustered with African individuals in the PCA), were genetically very similar despite being collected from different locations and/or in different years, as shown by their close clustering and minimal genetic distance in the NJ tree (Supplementary Figure S3).

### 3.3. Diagnostic SNP Set for Origin Tracing

**Figure 3:**
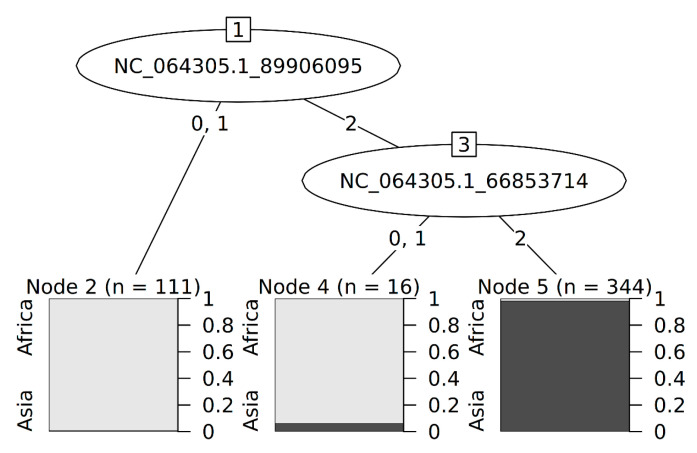
Decision tree using 2 *Bactrocera dorsalis* genomic SNPs (NC_064305.1:89906095 and NC_064305.1:66853714). The partitioning of the training samples between African samples in lightgrey and Asian samples in dark grey is indicated. 0, 1 and 2 represent the count of the reference allele for each SNP.

Recurrent feature elimination using a random forest approach achieved a maximum accuracy (success rate) of 0.9944 when 15 SNPs were used as predictors to infer the region of origin (SD = 0.01188, Cohen’s Kappa = 0.9851, Cohen’s Kappa SD = 0.03145), with accuracy plateauing beyond 15 SNPs (Supplementary Figure S4 and Supplementary Table S11). Robustness of the 15-SNP model was evaluated through 100 permutations, each time using a different subset of the 471 individuals (131 African, 340 Asian) as validation samples and achieved an accuracy of 0.9634% (SD = 0.0267) for African samples and 0.9995% (SD = 0.023) for Asian samples. Using a model consisting of only the two most informative SNPs (NC_064305.1:89906095 and NC_064305.1:66853714) resulted in an overall accuracy of 0.9546 (SD = 0.01188), with an accuracy of 0.9497 (SD = 0.0374) for African individuals, and 0.9944 (SD = 0.0679) for Asian samples (Figure 3).

The geographical origin of the Belgian *B. dorsalis* samples, including trapped adults (6 samples) and larvae intercepted from fruits (9 samples), was identified as either Asian or African. For seven individuals (5 trapped adults and 2 intercepted larvae), the origin could not be traced due to missing data, as the method requires all SNPs in the panel to be accurately genotyped (Table 2).

### 3.4. Origin Tracing via DAPC of COI DNA Barcodes

**Figure 4:**
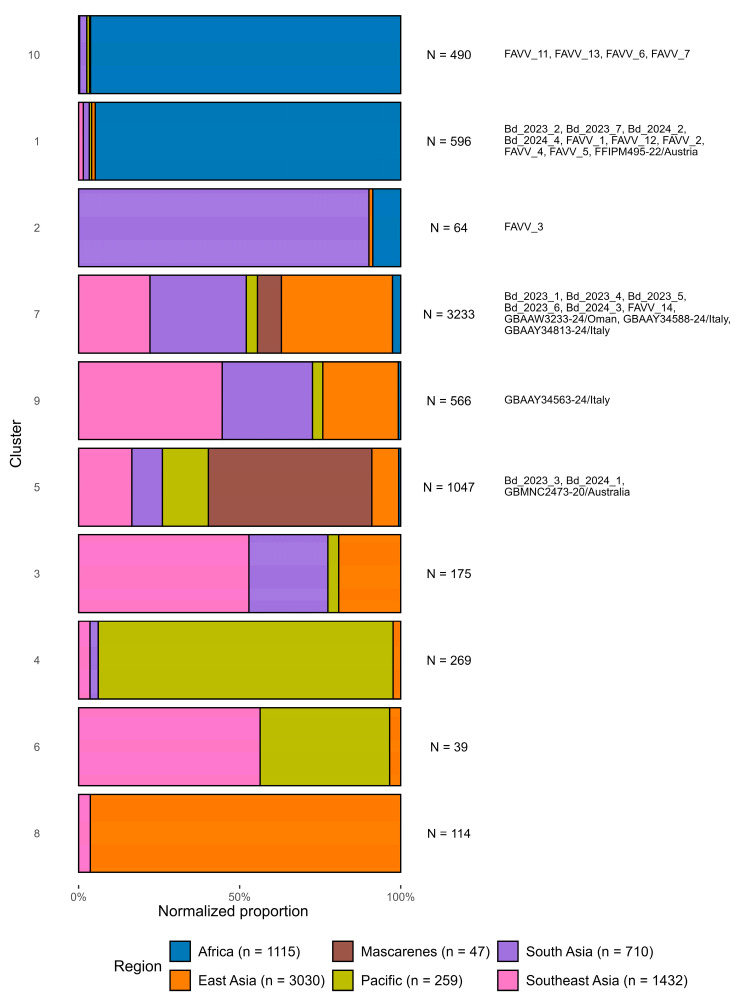
Assignment of the COI barcodes of trapped/intercepted *Bactrocera dorsalis* specimens in Belgium to DAPC clusters. Each horizontal bar represents one DAPC cluster. For each DAPC cluster, the total number of COI barcodes (N =) from *Bactrocera dorsalis* specimens with known geographic origin (excluding those specimens from insufficiently documented areas in Europe and West Asia) is displayed to the right of the respective bar (see also Supplementary Tables S12 and S13). Regional proportions within each cluster were normalized across six geographic regions (Africa, East Asia, South Asia, Southeast Asia, Mascarenes, Pacific), with the number of reference samples (*n* =) from each region indicated in parentheses. The assignments of the COI barcodes from the trapped/intercepted Belgian specimens and six additional *Bactrocera dorsalis* COI barcodes are shown on the utmost right of each DAPC cluster.

An alignment of 8404 COI DNA barcodes of length 684 bp was retained after filtering and trimming and used in DAPC to identify haplotype clusters (Supplementary Tables S12 and S13). We inferred ten haplotype clusters, containing 6593 COI DNA barcodes of *B. dorsalis* with known origin, which were used to calculate normalized regional proportions per DAPC cluster (Figure 4). Two clusters (1 and 10) were dominated by COI DNA barcodes of African specimens (94.8% in cluster 1, 96.3% in cluster 10) (Figure 4, Supplementary Table S13 and S14, Supplementary Figure S5). Clusters 2, 3, 7, 8, 9 predominantly include COI DNA barcodes from Asia. In this context, and following Deschepper et al. [54], the reference DNA barcodes from the Mascarenes, recovered in clusters 5 and 7, was also considered as representative of Asian profiles. The DNA barcodes from the Pacific Ocean region (Papua New Guinea, French Polynesia, United States) were largely assigned to cluster 4 and 6. Clusters 2 and cluster 4 displayed complex taxonomic and/or geographic patterns. Cluster 2, for example, contained a large proportion of COI DNA barcodes from specimens originating in South Asia (mostly Sri Lanka, Supplementary Table S12), but also included barcodes from African specimens. When the COI DNA barcodes of individuals from the WGS reference dataset assigned to this cluster—CDKB02, LKAD01, LKAD04, LKAD06, LKAD07, LKAD08—were queried in BOLD systems, several could not be reliably distinguished from *B. kandiensis*, another species within the *B. dorsalis* complex (employing a genetic distance cutoff of 1% for species discrimination; see Supplementary Table S15). Cluster 4, on the other hand, was predominantly composed of COI DNA barcodes from specimens from the Pacific region, including 81.6% of the 191 *B. dorsalis* specimens from Hawaii or intercepted in California, but it also contained barcodes from 22 of the 25 *B. carambolae* specimens from the WGS reference dataset. Lastly, none of the three South African individuals that clustered in the PCA with *B. dorsalis* individuals of Asian origin (ZAMP06, ZAMP07 and ZAMP09, Figure 2B) were assigned to one of the typical African haplotype clusters (cluster 1 or cluster 10).

The DAPC assigned the COI DNA barcode of all *B. dorsalis* specimens that were trapped/intercepted in Belgium (Bd_202## and FAVV_# samples) to a haplotype cluster with high confidence, with individual posterior probabilities ranging between 0.995 and 1.0. The COI DNA barcodes of four trapped *B. dorsalis* adults were assigned to cluster 1, which is dominated by reference specimens from Africa and therefore considered to reflect an African origin. For three of these samples—Bd_2023_2, Bd_2023_7, and Bd_2024_2—this was consistent with the origin assignment based on nuclear SNP data. In contrast, Bd_2024_4 was also assigned to this cluster but was identified as of Asian origin based on nuclear SNP data (Figure 2, Table 2). The remaining trapped *B. dorsalis* adults were assigned to either cluster 5 (Bd_2003_3, Bd_2004_1) or cluster 7 (Bd_2003_1, Bd_2003_4, Bd_2003_5, Bd_2003_6, Bd_2004_3) which, as described above, supports an Asian origin for these individuals. These results are consistent with the origin assignment based on nuclear SNP data, which also identified these individuals as of Asian origin.

Among the intercepted *B. dorsalis* larvae, DAPC assigned FAVV_7, FAVV_11, and FAVV_13 to cluster 10, while FAVV_1, FAVV_2, FAVV_4, FAVV_5, and FAVV_12 to cluster 1. As previously described, both clusters are dominated by African reference specimens, supporting the African origin for these larvae. Conversely, FAVV_3 was assigned to cluster 2, which predominantly comprises reference specimens from South Asia, with a minor representation of specimens from Africa. This result is incongruent with the nuclear SNP data, which unequivocally support an African origin for FAVV_3. Further, FAVV_14 was assigned to cluster 7, which, as described above, supports an Asian origin. and is consistent with geographic assignment of FAVV_14 based on nuclear SNP data.

In addition to the trapped/intercepted specimens in Belgium, several *B. dorsalis* DNA barcodes from non-endemic regions were included in the DAPC to explore potential origin profiles. These included publicly available accessions from Australia, Italy, and Oman, as well as one specimen from Austria (FFIPM495-22) recovered in the context of the Fruit-Flies In Silico Precision & Management (FF-IPM) project (https://fruitflies-ipm.eu/). As *B. dorsalis* is not known to have established populations in these countries at the time of publication, their inclusion serves to provide broader context on potential invasion dynamics. Three specimens, GBAAW3233-24 (Oman), GBAAY34588-24 and GBAAY34813-24 (Italy), were assigned to cluster 7, which, as previously is therefore interpreted as indicative of an Asian origin. GBMNC2473-20 from Australia was assigned to cluster 5, which also reflects an Asian profile. GBAAY34563-24 (Italy) was assigned to cluster 9, which comprises reference specimens predominantly from Asia. Conversely, specimen FFIPM495-22 from Austria was assigned to cluster 1, and was therefore interpreted as being of African origin.

Lastly, a user-friendly R-script that assigns a COI barcode of *B. dorsalis* to its corresponding DAPC cluster, thereby offering an indication of its geographic origin, was included as a supplementary file (Supplementary File S1).

### 3.5. Phylogenetic Analysis of Mitogenomes

**Figure 5:**
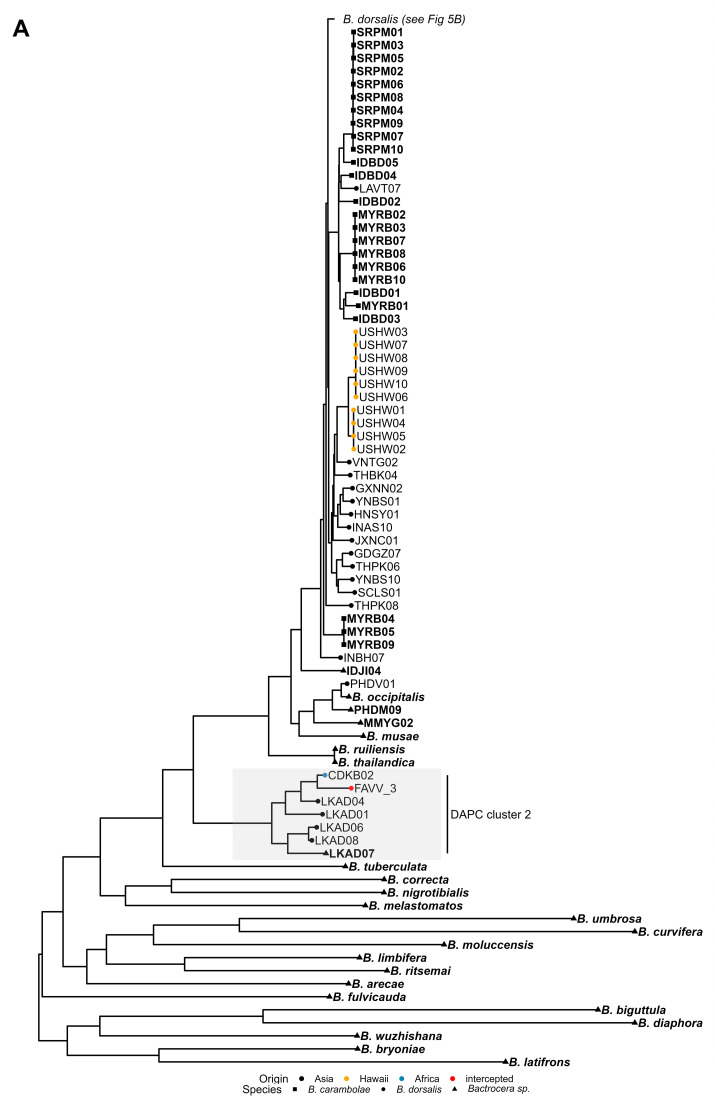

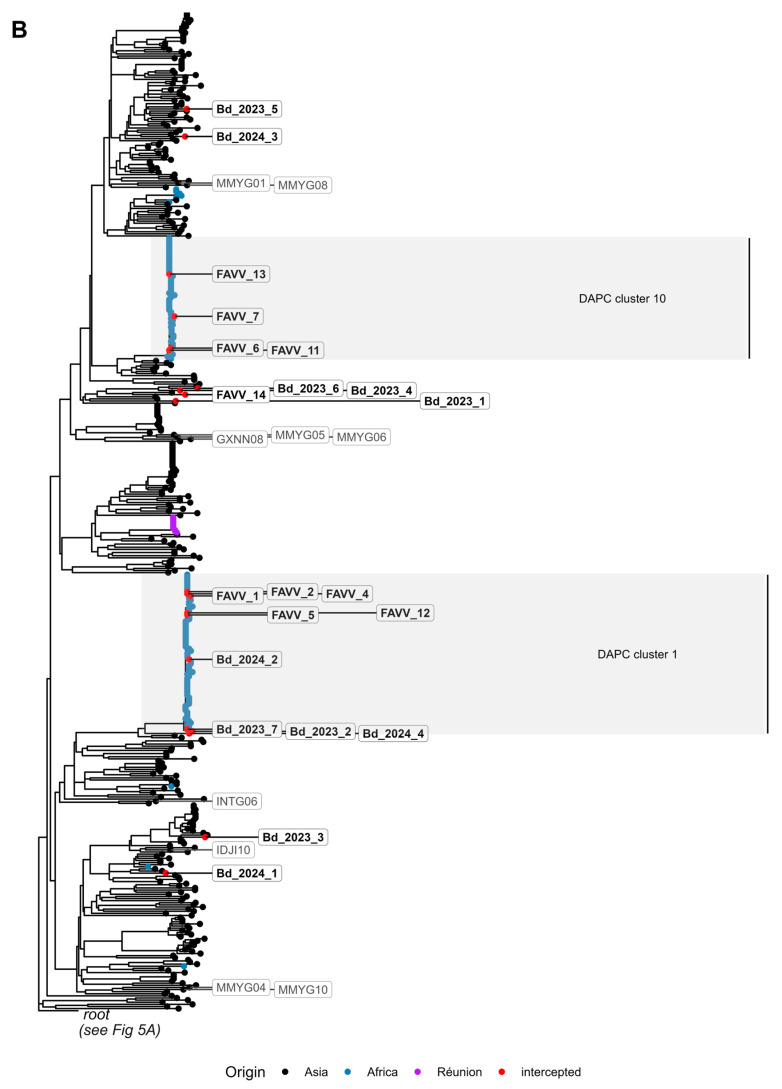
Maximum likelihood mitogenomic tree reconstructed with FastTree. The phylogenetic tree was split into two figure panels, with the root of the tree shown in panel (**A**), and the main *Bactrocera dorsalis* branch showing in panel (**B**). (**A**) *Bactrocera dorsalis* individuals that clustered with either the African or Asian group in the PCA are labelled with regular font, while *Bactrocera carambolae* individuals and outliers in the PCA are shown in bold, non-italicized font (see Figure 2). *Bactrocera dorsalis* and *Bactrocera carambolae* individuals are indicated with a dot and a square at the branch tip, respectively. Individuals from other species or outliers in the PCA are indicated with a triangle. Branch tip colour of *Bactrocera dorsalis* individuals indicates their (country of) origin or is red for individuals trapped/intercepted in Belgium. The clade corresponding with DAPC cluster 2 is highlighted. (**B**) * Bactrocera dorsalis* individuals that clustered with either the African or Asian group in the PCA are unlabeled, except for individuals trapped/intercepted in Belgium (framed labels with bold black font). PCA outliers have framed labels with grey font (see Figure 2). Branch tip colour of *Bactrocera dorsalis* individuals indicates their (country of) origin or is red for individuals trapped/intercepted in Belgium. Clades corresponding with DAPC clusters 1 and 10 were highlighted. For bootstrap support of nodes, see Supplementary Figure S6.

Following alignment of the 562 mitogenome sequences, eleven entries were excluded due to excessive missing data or low sequence quality. This included one reference genome (*B. rubigina,* NC_046521.1), several WGS reference dataset samples (MMYG07, and all but two individuals from the YNJH sample) and one Réunion sample, all exhibiting a high proportion of undetermined bases. A total of 551 mitogenomes (Supplementary Table S5 and Supplementary File S2) were retained for phylogenetic inference (Figure 5, Supplementary Figure S6, Supplementary File S3).

Consistent with the findings of Doorenweerd et al. [47], the phylogenetic analysis of mitogenomes did not recover a single monophyletic clade encompassing all specimens morphologically identified as *B. dorsalis* (Figure 5, Supplementary Figure S6). Instead, the resulting topology revealed a more complex structure comprising three distinct groups. The first supported group consisted of five *B. dorsalis* specimens from Sri Lanka (LKAD01, LKAD04, LKAD06, LKAD07, LKAD08), one from the Democratic Republic of Congo (CDKB02), and FAVV_3, a larva intercepted from imported African fruit. All individuals from this group were assigned to cluster 2 in the DAPC analysis (see Section 3.4). As previously noted, these specimens yielded ambiguous *B. dorsalis*—*B. kandiensis* COI DNA barcoding identifications (see Section 3.4). Noteworthy, the LKAD08 specimen of this group was recovered as an outlier in the PCA based on nuclear SNP frequencies (see Section 3.2). The second supported group was a basal polyphyletic clade, containing *B. dorsalis* specimens MMYG02, PHDM09 and PHDV01, alongside a representative of *B. occipitalis* and *B. musae*. Of particular note, PHDM09 and MMYG02 were also recovered as outliers in the PCA based on nuclear SNP frequencies (see Section 3.2). The third and largest group comprised almost all *B. dorsalis* specimens from Asian and African origin as well as *B. carambolae* representatives, with the latter forming multiple clades intermixed with *B. dorsalis*. As in the other two groups, this third group also contained several individuals of the WGS reference dataset (GXNN08, INTG06, IDJI04, IDJI10, MMYG01, MMYG04, MMYG05, MMYG06, MMYG08 and MMYG10) that were outliers in the PCA based on nuclear SNP frequencies (see Section 3.2). Notably, the three South African individuals that grouped with *B. dorsalis* of Asian origin in the PCA (ZAMP06, ZAMP07, and ZAMP09; Figure 2B) also clustered within this third group, and within clades composed almost exclusively of mitogenomes from Asian individuals (Supplementary Figure S6).

Importantly, although a considerable proportion of the *B. dorsalis* subclades in the phylogenetic tree comprised samples from the same geographic regions (e.g., Hawaii, Réunion), no two clearly distinct clades corresponding to African and Asian populations were observed, as would be expected under a scenario exhibiting a strong and consistent phylogeographic signal. Instead, the presence of multiple exceptions, most notably, six African *B. dorsalis* specimens (MLBM02, MLBM06, CIBD04, GHND05, NGNS03 and CDKB05) clustering within subclades primarily composed of Asian samples, complicated a straightforward interpretation of geographic structuring. However, for the purposes of this study, the geographic origin of trapped/intercepted specimens was inferred based on the origin of individuals of the WGS reference dataset clustering in close phylogenetic proximity. With the exception of FAVV_3, the mitogenomes of trapped/intercepted *B. dorsalis* specimens (Bd_202##_# and FAVV_# samples) clustered within clades composed of mitogenomes from either African or Asian *B. dorsalis* specimens. For all larvae, excluding FAVV_3, the inferred geographic origins matched the known origin. Similarly, for all adults, except Bd_2024_4, the results were consistent with both DAPC- and SNP-based origin assignments (Table 2). Remarkably, the mitogenome of individuals within one *B. dorsalis* pair in the NJ tree based on nuclear SNP frequencies (Bd_2024_1/Bd_2024_4, Supplementary Figure S3) clustered into different subclades in the maximum likelihood phylogenetic analysis (Figure 5, Figure S6, Supplementary File S3), while the mitogenomes of the other pair both clustered within the same subclade (Bd_2023_7/Bd_2024_2, Supplementary Figure S3). Pairwise alignment of the mitogenomes (Supplementary File S4) revealed a 99.9% identity between Bd_2023_7 and Bd_2024_2, and a 99.1% identity between Bd_2024_1 and Bd_2024_4.

## 4. Discussion

### 4.1. Genomic Surveillance Uncovers Diverse Origins of B. dorsalis in Belgium

Between 2023 and 2024, eleven adult males of *B. dorsalis* were trapped in Belgium. Based on a PCA of nuclear SNP frequencies, two adults trapped at Anderlecht had an African origin, while nearly all adults trapped in Antwerp, with the exception of Bd_2023_7, exhibited an Asian origin. In contrast to the trapped adults, the majority of the *B. dorsalis* larvae intercepted in Belgium, except FAVV_14, were assigned an African origin. The latter aligns with the observation that the majority of *B. dorsalis* larvae intercepted at Belgian border inspection posts are found in mangoes imported from Africa [18].

The differing origins of the trapped *B. dorsalis* adults show that these individuals arrived via multiple, likely independent introduction pathways. While direct introductions from regions outside the EU, particularly Africa and Asia, remain a primary hypothesis, the data also point to more complex scenarios involving internal movement within the EU. One plausible route involves indirect introductions, that is, from fruits and vegetables of African or Asian origin imported within other EU countries and subsequently transferred to Belgium via authorized trade, which, under current phytosanitary regulations, is not systematically subjected to official inspection. A brief review of mango imports in Belgium and neighbouring countries over the past two years indicates that such a scenario is likely: imports (expressed in kg) from Asian countries where *B. dorsalis* occurs were 24- and 57-fold higher in the Netherlands and Germany than in Belgium. Furthermore, approximately 25% of Belgium’s mango imports originated from the Netherlands, the main European trade hub for mangoes (Supplementary Table S16, [55,56]). Consequently, the presence of *B. dorsalis* adults of Asian origin in Belgium may plausibly be explained by the importation of mangoes originating from Asia and re-exported through the Netherlands or Germany, rather than by direct introductions from Asian regions. Additionally, unauthorized trade and informal exchanges, such as personal transport of fruits by travellers, represent another underestimated pathway. These movements, whether from non-EU countries or within the EU, are hardly monitored and can contribute significantly to the spread of quarantine pests like *B. dorsalis* [3].

A particularly intriguing possibility is that some of the Belgian interceptions may have originated from transient populations within the EU, such as those previously reported in Italy [19,20,24], from repeated incursions in neighbouring countries like France [22,23], or worse, from undetected established Belgian populations. These introductions could reflect a broader pattern of regional dispersal, potentially involving multiple stepping-stone events. Unfortunately, verifying the first two hypotheses is currently not possible, as WGS data from the French incursions or Italian (transient) populations is not available. However, the alternative explanation of an undetected, established population in Belgium appears unlikely, since *B. dorsalis* is currently unable to survive Belgian winters, which include at least five days with a maximum air temperature below 0 °C [57,58,59]. Furthermore, while two pairs of trapped *B. dorsalis* adults were observed in the NJ tree based on nuclear SNP frequencies—suggesting a shared source population for each pair—their mitogenomes are not entirely identical, and the mitogenomes of individuals of one pair clustered into different subclades in the phylogenetic analysis. As mitochondrial genomes are maternally inherited, this further reduces the likelihood of an established population in Belgium.

### 4.2. A SNP Based Diagnostic Framework for Origin Tracing of B. dorsalis

A perceived major drawback of WGS is the relatively high cost associated with achieving sufficient depth of coverage, and one might consider a targeted genome sequencing approach instead. For example, Charbonnel et al. [60] employed restriction site-associated DNA sequencing (RAD-seq) to resolve the genetic structure of 68 *B. dorsalis* individuals from countries in native Asia, Africa, and islands in the Indian and Pacific Oceans. Although WGS is generally expected to offer higher resolution, their findings indicated that RAD-seq was equally effective in resolving global geographic patterns. They further estimated that using WGS would have tripled the cost of their study.

Alternatively, origin tracing can also be achieved by using a limited set of genome-wide diagnostic markers. Over recent years, a variety of strategies have been employed to detect and extract diagnostic SNP loci for population assignment. For example, Fst-based pruning has proven effective in developing diagnostic SNP panels for origin tracing of the Mexican fruit fly (*Anastrepha ludens*, [61]) and the Spanish Cedar (*Cedrela odorata*, [62]). This study evaluated the effectiveness of random forest machine learning algorithms in identifying nuclear SNPs that are highly informative for determining the geographic origin of trapped or intercepted *B. dorsalis* specimens. Preliminary in silico analyses demonstrated that even a minimal set of the 15 most informative SNPs could achieve a mean assignment accuracy exceeding 96% for regional origin. Remarkably, reducing the panel further to just two SNP loci was still sufficient to distinguish between the two major continental regions of origin, Africa and Asia, with a mean accuracy of almost 95%. While further subdivision to resolve lower-level genetic clusters (e.g., country or subregional origins) is appealing, it introduces trade-offs: finer-scale resolution requires more intensive sampling and can reduce the robustness of the model due to smaller sample sizes for each group. Random forest analysis is particularly well-suited for these classification tasks, as it can efficiently handle large genomic datasets and identify predictive features even when diagnostic loci are sparse. However, achieving more resolved geographic resolution (e.g., within continents or regions) would necessitate extensive regional sampling and validation, which may not always be feasible.

The reference database used to train the diagnostic model includes a wide representation of *B. dorsalis* populations from across its global range, supporting its expected robustness when applied to specimens intercepted from non-EU regions. This is exemplified by the correct assignment of the FAVV_# samples, larvae intercepted in fruit imports, according to the country of origin of the fruit, even though no training samples from those specific populations were included. These results suggest that the model can generalize effectively across geographically diverse populations. However, its performance on potential interceptions originating from within the EU remains uncertain due to the lack of sequencing data for the reference samples from the transient population in Italy or from other possible sources within the EU, including France, where adult *B. dorsalis* has also been trapped [19,20,22,23,24]. RAD-sequencing of these samples suggested that the Italian transient populations all originate from continental Asia, whereas incursions from France were primarily from West Africa, but also from mainland Asia and Réunion [63]. Unfortunately, these data are not publicly available and it is unclear whether the RAD-sequencing approach encompasses the diagnostic SNP set reported in this study. Therefore, incorporating WGS data from these populations into the reference dataset will be essential in the future to fully assess and potentially improve the model’s diagnostic accuracy in the European context.

A minimal SNP set that enables robust discrimination between the African and Asian origins of *B. dorsalis* paves the way for rapid and cost-effective diagnostic assays, such as targeted SNP genotyping (e.g., probe-based qPCR, digital droplet PCR or KASP assays [64,65]). Such assays would enable fast, informed responses to *B. dorsalis* incursions, both in regions where the species has already been detected (e.g., Belgium and France) and in those at high risk of introduction (e.g., Spain or Portugal [66]), thereby avoiding delays from sample sequencing and complex bioinformatic analyses. While this approach does not pinpoint the exact country or region of origin, it still provides valuable information. Determining whether a trapped/intercepted specimen likely originates from Africa or Asia can help narrow down potential introduction pathways and guide follow-up investigations. For instance, repeated interceptions from a particular continent could prompt authorities to scrutinize associated trade routes, commodities, or passenger flows and adjust inspection protocols accordingly.

As part of a continued effort to improve diagnostics and support responses to new incursions, the SNP-based diagnostic approach described in this study is currently being adapted to increase its resolution while maintaining cost-efficiency. For example, to detect origins from the regional groups described by Zhang et al. [27], including China, Northern Southeast Asia, Southern Southeast Asia, Indian Subcontinent, Africa, Hawaii, or by Charbonnel et al. [60], who described similar patterns and could also resolve, to some extent, East from West African populations. Emerging ortholog-based phylogenomic approaches, which have shown promising results in the *Anastrepha fraterculus* species complex [67,68], could also be explored within the random forest framework to enhance regional resolution and SNP diagnostic power. Such integration may help disentangle the mixed phylogenetic and phylogeographic signals in *B. dorsalis*, potentially reducing the reliance on additional extensive sampling.

### 4.3. DNA Barcoding as a Shortcut: Cost- and Time-Effective Origin Tracing with Imperfect Data

In addition to nuclear SNPs, this study also assessed whether a mitochondrial DNA fragment (COI DNA barcode) could be used for origin tracing of *B. dorsalis*. COI DNA barcoding is frequently used for identification of animal species [69]. However, for the *B. dorsalis* complex it does not consistently provide sufficient discriminatory power to distinguish *B. dorsalis* from other members, including *B. carambolae*, *B. kandiensis*, *B. occipitalis*, and *B. raiensis*, within the complex [12,13,48]. This lack of resolution is primarily due to the shallow phylogenetic structure within the complex, further complicated by incomplete lineage sorting and interspecific hybridization [7,49]. Moreover, the large volume of publicly available reference data is inevitably associated with some degree of mislabeling or misidentification, particularly in this group, which has historically been characterized by unstable and conflicting taxonomic classifications [46]. This further obscures molecular identifications and undermines the reliability of DNA barcoding in this context.

Rather than relying on fixed haplotype differences, as in “classical” DNA barcoding identification [70], a frequency-based approach was applied using DAPC on available COI barcode data. This method leverages differences in haplotype distributions across regions, allowing for probabilistic inference of origin even when haplotypes are widely shared [71]. Interestingly, despite all the caveats, DAPC of COI barcodes performed reasonably well as a screening tool for origin tracing. Most African samples clustered distinctly, and the Mascarene population, though geographically close to Africa, exhibited a genetic signature closely aligned with Asian populations, a result consistent with broader genomic analyses [54,60]. Moreover, the geographic assignments produced by DAPC of COI barcodes were generally in agreement with those obtained from the more robust SNP-based system, both for adults and larvae trapped/intercepted in Belgium.

However, the DAPC assigned FAVV_3—one of the *B. dorsalis* larvae intercepted from imported African fruit and determined to have an African origin based on nuclear SNP frequencies—to DAPC cluster 2, which was dominated by specimens of Asian origin. The phylogenetic analysis of mitogenomes confirmed this result, placing FAVV_3 within a small clade of six individuals (five from Asia and one from Africa) belonging to DAPC cluster 2. The COI sequences from members of this clade also produced ambiguous identifications, falling between *B. dorsalis* and *B. kandiensis*, with the latter species being reported as endemic to Sri Lanka [72]. In this context, it is challenging to disentangle signals of biogeographic differentiation from those of potential interspecific hybridization. The latter may also be observed in the largest group of the phylogenetic tree, where *B. dorsalis* mitogenomes cluster with those of *B. carambolae*, and is in line with previous reports of mitochondrial introgression involving *B. dorsalis*, *B. carambolae*, and *B. kandiensis* [13,47]. Furthermore, in this largest group, mitonuclear discordance was also observed for six African *B. dorsalis* individuals from the WGS reference dataset, with all individuals having mitogenomes that cluster with those of Asian individuals. Conversely, Bd_2024_4, a trapped *B. dorsalis* adult, clustered with African mitogenomes, whereas the PCA placed it among specimens of Asian origin. Consequently, it is concluded that the mitochondrial markers used in this study, both the relatively short COI DNA barcode and the full mitochondrial genome, while yielding reliable origin tracing for the vast majority of *B. dorsalis* samples, cannot always provide conclusive evidence of geographic origin.

## 5. Conclusions

Eleven adult *B. dorsalis* males were trapped in Belgium between August 2023 and November 2024. A PCA based on nuclear SNPs assigned these specimens to either African or Asian origins. To enable rapid and cost-effective origin tracing, a diagnostic set of two nuclear SNPs and a DAPC based on mitochondrial COI barcodes was developed. The former approach showed high accuracy in distinguishing African from Asian lineages, while the DAPC was less reliable, particularly in cases of mitonuclear discordance. Nevertheless, both methods indicated multiple independent introductions of *B. dorsalis* into Belgium, with most trapped adults originating from Asia. This contrasts with the predominantly African larvae found in imported fruit and suggests that the adults likely entered via alternative routes—such as intra-EU trade or unauthorized fruit imports—highlighting the need for improved monitoring of fruit and vegetable trade within the EU.

## Figures and Tables

**Table 2:** Inference of geographic origin for the interceptions of *Bactrocera dorsalis* in Belgium. (1) visual inspection of PCA and NJ tree (PCA/NJT) reconstructed from the covariance matrix of nuclear allele frequencies, (2) random forest-based decision tree based on two nuclear SNPs (3) DAPC of COI DNA barcodes. For each method, specimens were profiled as of African or Asian origin. “NA” refers to individuals that could not be assigned a geographic origin due to missing genotype data.

Specimen	PCA/NJT	SNPs		COI DAPC			Consensus
	Inferred Origin	Inferred Origin	Prob.	Haplotype Cluster	Posterior Prob.	Inferred Origin	
Bd_2023_1	Asia	NA	NA	7	1.0000	Asia	yes
Bd_2023_2	Africa	NA	NA	1	1.0000	Africa	yes
Bd_2023_3	Asia	NA	NA	5	1.0000	Asia	yes
Bd_2023_4	Asia	Asia	1.0000	7	1.0000	Asia	yes
Bd_2023_5	Asia	NA	NA	7	1.0000	Asia	yes
Bd_2023_6	Asia	Asia	1.0000	7	1.0000	Asia	yes
Bd_2023_7	Africa	Africa	1.0000	1	1.0000	Africa	yes
Bd_2024_1	Asia	Asia	1.0000	5	1.0000	Asia	yes
Bd_2024_2	Africa	Africa	1.0000	1	1.0000	Africa	yes
Bd_2024_3	Asia	NA	NA	7	1.0000	Asia	yes
Bd_2024_4	Asia	Asia	1.0000	1	0.9954	Africa	no
FAVV_1	Africa	Africa	1.0000	1	1.0000	Africa	yes
FAVV_2	Africa	NA	NA	1	1.0000	Africa	yes
FAVV_3	Africa	Africa	1.0000	2	1.0000	Africa or South Asia	no
FAVV_4	Africa	Africa	0.9357	1	1.0000	Africa	yes
FAVV_5	Africa	NA	NA	1	1.0000	Africa	yes
FAVV_6	Africa	Africa	1.0000	10	1.0000	Africa	yes
FAVV_7	Africa	Africa	1.0000	10	1.0000	Africa	yes
FAVV_11	Africa	Africa	1.0000	10	1.0000	Africa	yes
FAVV_12	Africa	Africa	1.0000	1	1.0000	Africa	yes
FAVV_13	Africa	Africa	1.0000	10	1.0000	Africa	yes
FAVV_14	Asia	Asia	1.0000	7	1.0000	Asia	yes

## Data Availability

The bioinformatic pipelines described in this study are deposited at https://github.com/PESTFLY/PESTFLY_Insects_2025. Newly generated DNA sequencing data was deposited in NCBI GenBank under accession number PRJNA1254113.

## Supplementary Materials

The following supporting information can be downloaded at: https://www.mdpi.com/article/10.3390/insects16121271/s1, Figure S1: Selection of the optimal number of genetic clusters (K) of COI-5P haplotype variation using the diffNgroup criterion in the find.clusters function (adegenet); Figure S2: Scree plot indicating the relative importance of the first 10 principal components from the nuclear allele frequency variation; Figure S3: Neighbour-joining (NJ) clustering based on the covariance matrix of nuclear allele frequencies; Figure S4: Recursive feature elimination identifies the optimal number of SNPs for accurate classification; Figure S5: Geographic distribution of COI-5P haplotype clusters across regions (A) and countries (B); Figure S6: Mitogenomic haplotype tree constructed with FastTree; Table S1: FASFC *B. dorsalis* sample details; Table S2: Metadata of populations used for the WGS reference dataset from Zhang et al. [27]; Table S3: Metadata of individual specimens used for the nuclear allele frequency analysis; Table S4: *B. dorsalis* COI DNA barcodes downloaded from the BOLD Systems database; Table S5: Metadata of individual specimens used for the whole-mitogenome analysis; Table S6: Raw sequencing read quality metrics for data newly generated for this study; Table S7: Bam file mapping statistics for the original bam files used for the genome-wide nuclear allele frequency; Table S8: samtools flagstat statistics for the downsampled bam files used for the genome-wide nuclear allele frequency analysis; Table S9: Individuals from the WGS reference dataset not clustering with the two main *B. dorsalis* groups in the PCA; Table S10: Report of nuclear variant statistics per sample generated with bcftools stat; Table S11: Recursive feature selection identified a minimal subset of 15 SNPs; Table S12: Filtering of COI barcodes; Table S13: COI barcodes and their assigned DAPC cluster (with posterior probabilities); Table S14: Country-level counts of DAPC COI cluster assignments; Table S15: BOLD identification results for individuals of the WGS reference dataset assigned to COI DAPC Cluster 2; Table S16: Import from guavas, mangoes, mangosteens (HS code 080450) in Belgium, neighbouring countries, Italy and Spain, averaged over 2023 and 2024. File S1: R-script predict_supplement.R to assign a COI barcode to a DAPC- cluster, including an example (with 3 queries) to test the script. File S2: Alignment of *Bactrocera* sp. mitochondrial genomes used in phylogenetic analysis; Supplementary File S3: Phylogenetic tree of *Bactrocera* sp. mitochondrial genomes; File S4: Alignment of mitogenomes from four trapped *B. dorsalis* adults that closely clustered in the NJ tree based on nuclear SNP frequencies (see Figure S3).

## Abbreviations

The following abbreviations are used in this manuscript:

COI	Cytochrome Oxidase subunit I
SNP	Single-Nucleotide Polymorphism
GL	Genotype Likelihood
DAPCs	Discriminant Analysis of Principal Components
PCA	Principal Component Analysis
NPPO	National Plant Protection Organisation
FASFC	Federal Agency for the Safety of the Food Chain
WGS	Whole-Genome Sequencing
NJ	Neighbour-Joining
