# Supplementary material for: Genomic Tracing Reveals Multiple Independent Occurrences of Bactrocera dorsalis in Belgium
Source: Insects. 2025 Dec 15;16(12):1271. doi: 10.3390/insects16121271 (PMC12734248; doi:10.3390/insects16121271)
Supplement: Supplementary file 1 [file insects-16-01271-s001.zip › insects-3760316-supplementary/Supplementary Material/Supplementary_Figures_Vanbergen_et_al_2025_Insects.pdf]

Genomic tracing reveals multiple independent occurrences of Bactrocera dorsalis in Belgium

Sam Vanbergen[1,2], Pablo Deschepper[1,3], Jan Van Autreve[4], Vera Huyshauwer[4], Massimiliano Virgilio[1], Jochem Bonte[5], Wannes Dermauw[5]

1 Biology Department, Royal Museum for Central Africa (RMCA), Leuvensesteenweg 13, 3080 Tervuren, Belgium; sam.vanbergen@africamuseum.be
2 Ecology, Evolution and Conservation Biology, Biology Department, KU Leuven, 3000 Leuven, Belgium; sam.vanbergen@kuleuven.be
3 Biology Department, Royal Belgian Institute for Natural Sciences (RBINS),Vautierstreet 29 1000 Brussels, Belgium; pdeschepper@naturalsciences.be
4 Federal Agency for the Safety of the Food Chain (FASFC), Kruidtuinlaan 55, 1000 Brussel, Belgium
5 Flanders Research Institute for Agriculture, Fisheries and Food (ILVO), Plant Sciences Unit, Burgemeester Van Gansberghelaan 96, 9820, Merelbeke, Belgium; wannes.dermauw@ilvo.vlaanderen.be
* Correspondence: sam.vanbergen8@gmail.com (S.V.), wannes.dermauw@ilvo.vlaanderen.be (W.D)

Supplementary Figures

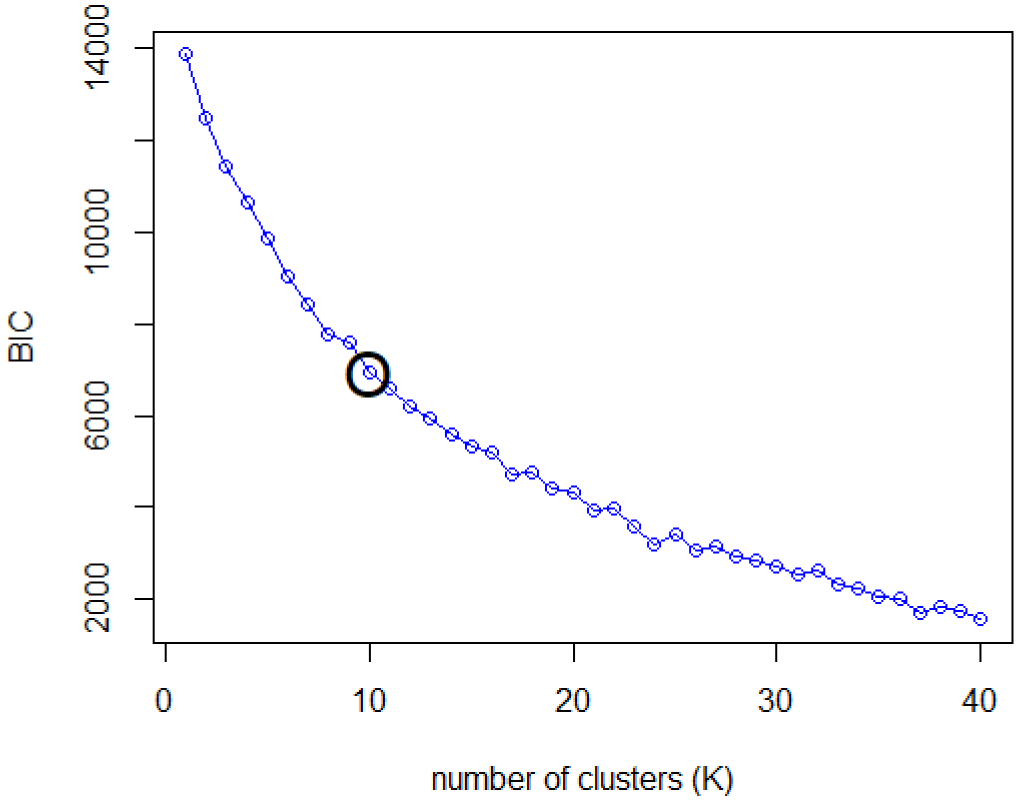

Supplementary Figure S1 - Selection of the optimal number of genetic clusters (K) of COI-5P haplotype variation using the diffNgroup criterion in the find.clusters function (adegenet). The Bayesian Information Criterion (BIC) values across a range of K values. The optimal number of clusters (K = 10) was selected at the point where the improvement in explanatory power began to plateau, as determined by the diffNgroup criterion (highlighted with a black circle).

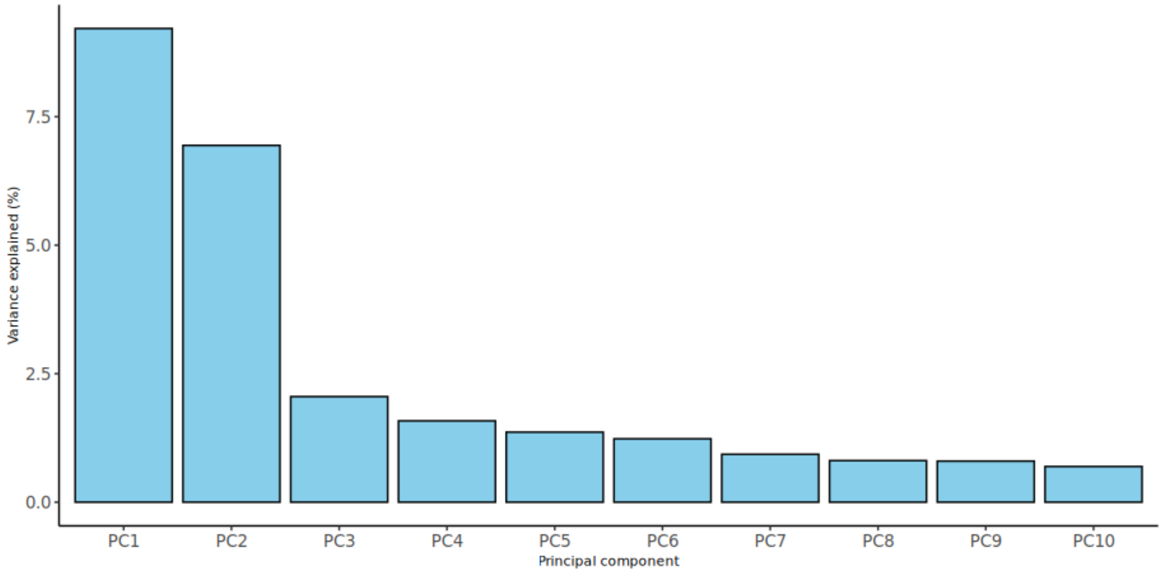

Supplementary Figure S2 - Scree plot indicating the relative importance of the first 10 principal components from the nuclear allele frequency variation.

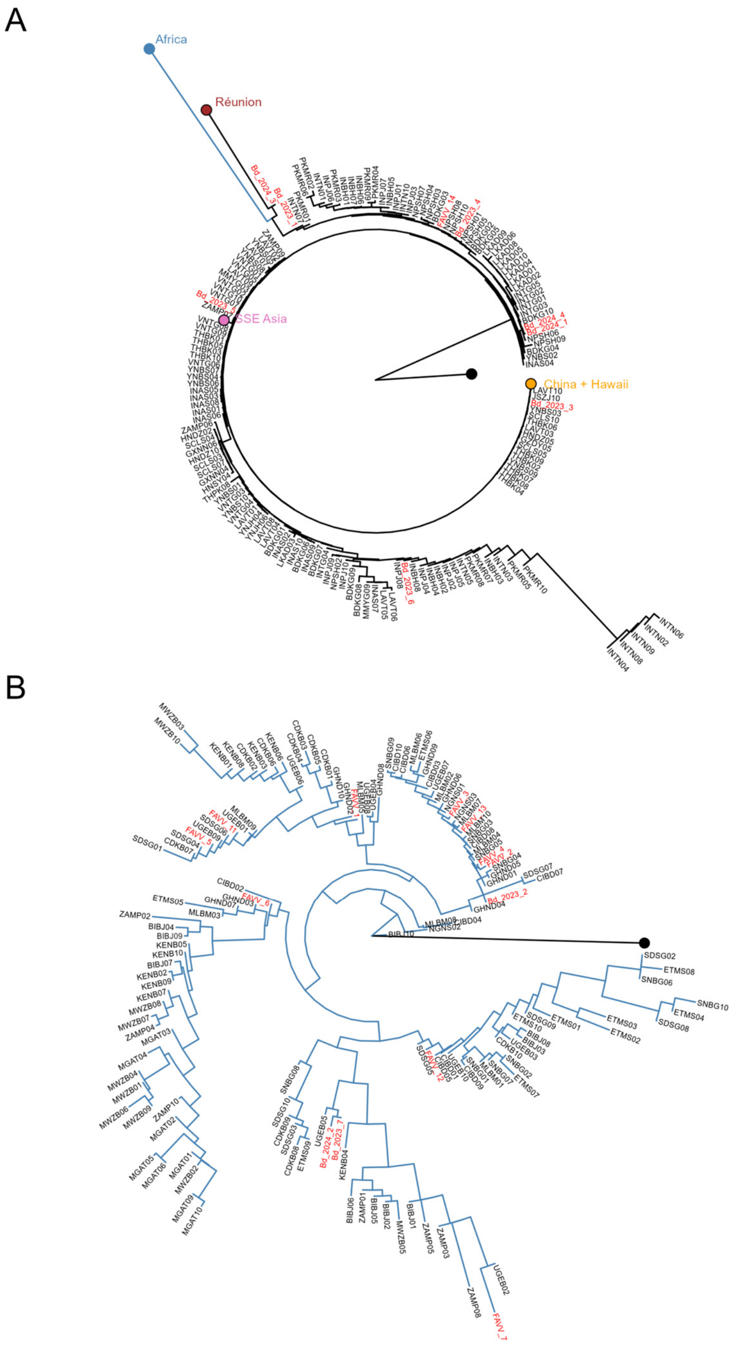

Supplementary Figure S3 - Neighbour-joining (NJ) clustering based on the covariance matrix of nuclear allele frequencies. This figure is presented as a complementary visualization of the genetic patterns highlighted by PCA in Figure 2. It provides an exploratory analysis of genetic relatedness within and across trapped/intercepted and reference specimens. *B. dorsalis* adults (Bd_202# samples) and larvae (FAVV# samples) trapped/intercepted in Belgium are labeled in red. For clarity, the tree was divided into two subtrees (A) the black dot represents PCA outliers and *B. carambolae* specimens in Figure 2A; the blue dot corresponds to specimens from Africa; the orange dot to specimens from China and Hawaii; the dark red dot to specimens from Réunion; and the pink dot to specimens from Southeast Asia. Two *B. dorsalis* adults that were trapped in Belgium (Bd_2024_1 and Bd_2024_4) clustered together. (B) uncollapsed view of the African cluster (blue dot in panel A), with all other groups collapsed (black dot). Two *B. dorsalis* adults that were trapped in Belgium (Bd_2023_7 and Bd_2024_2) clustered together.

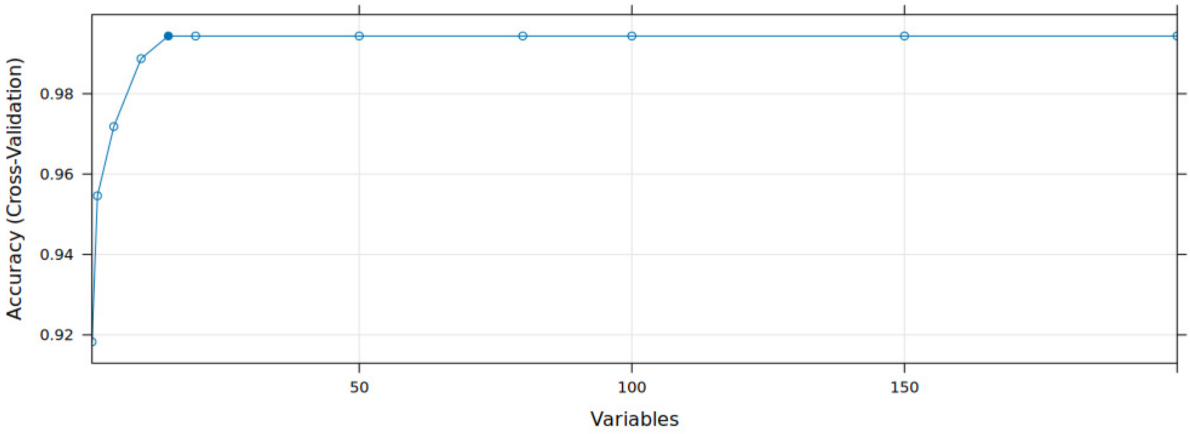

Supplementary Figure S4 - Recursive feature elimination identifies the optimal number of SNPs for accurate classification. Cross-validation accuracy as a function of the number of SNPs included in the model. Accuracy increases rapidly with the first few variables and plateaus after ~15 SNPs (filled blue dot), indicating that a small subset of markers is sufficient for high-confidence sample classification.

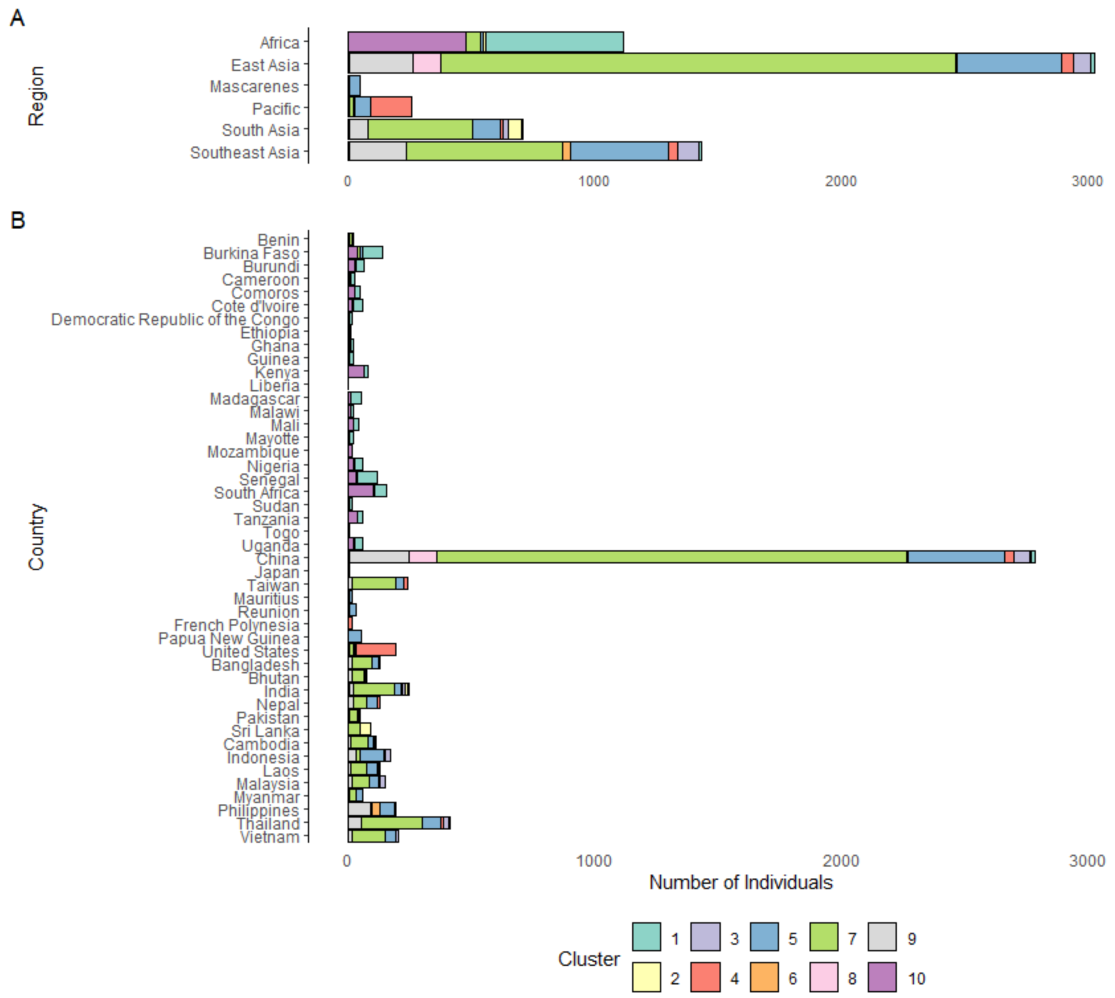

Supplementary Figure S5 - Geographic distribution of COI-5P haplotype clusters across regions (A) and countries (B). Stacked barplots show the number of individuals assigned to each DAPC-inferred COI cluster (color-coded) across broad geographic regions (A) and specific countries (B). Cluster 10 dominates in several African regions, while Asia exhibits greater cluster diversity. These patterns reflect geographic structuring of mitochondrial haplotype variation.

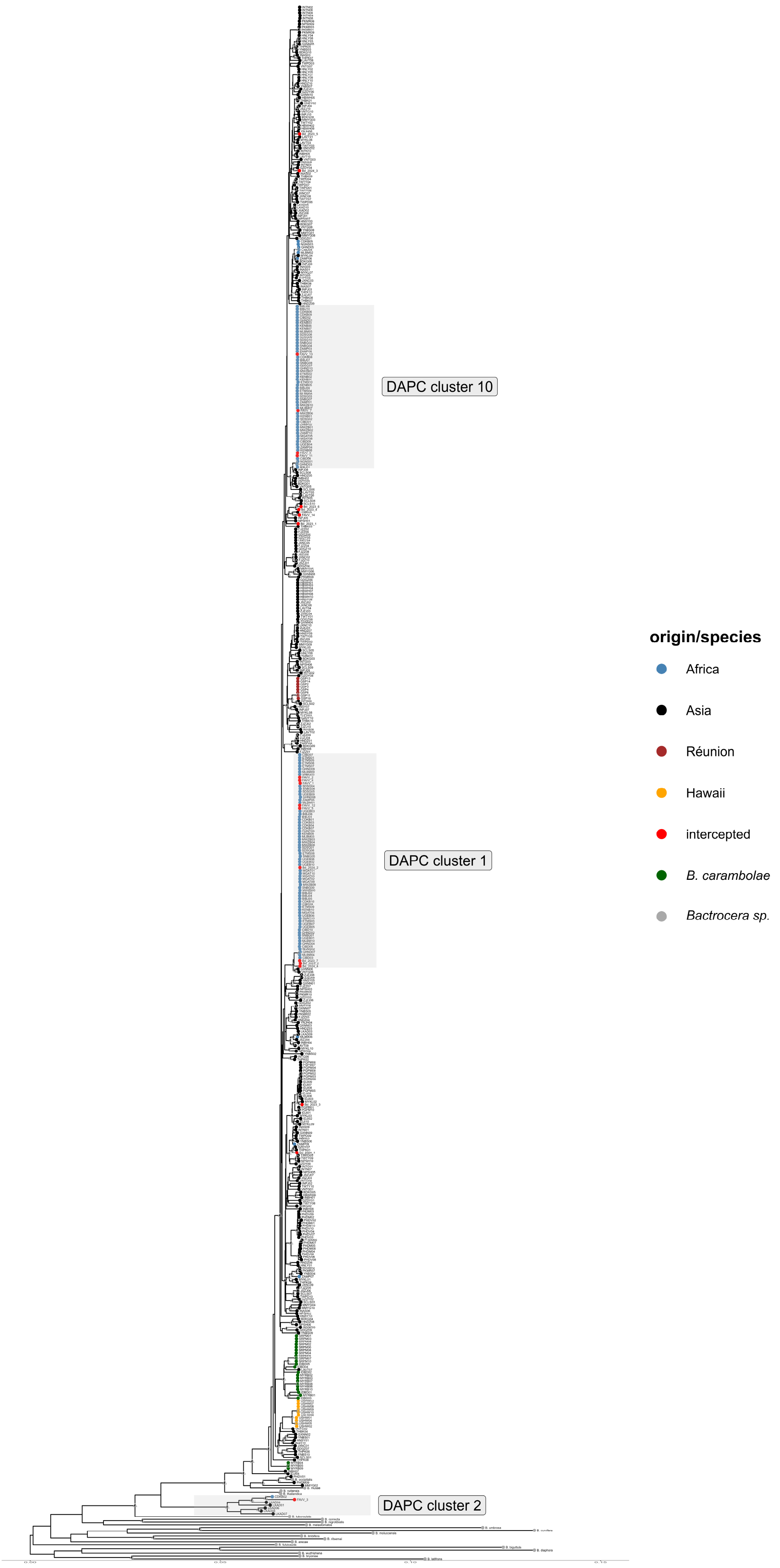

Supplementary Figure S6 - Mitogenomic haplotype tree constructed with FastTree. Same figure as Figure 5 showing hidden labels. All tips are labeled with their sample id, or species id in the case for NCBI RefSeq accessions. Clades corresponding with inferred COI haplotype clusters 1,2 and 10 were highlighted. Nodes supported by more than 95% of bootstraps are indicated with a triangle.
