# Supplementary figures and images for: Genomic Tracing Reveals Multiple Independent Occurrences of Bactrocera dorsalis in Belgium
Source: Insects. 2025 Dec 15;16(12):1271. doi: 10.3390/insects16121271 (PMC12734248; doi:10.3390/insects16121271)

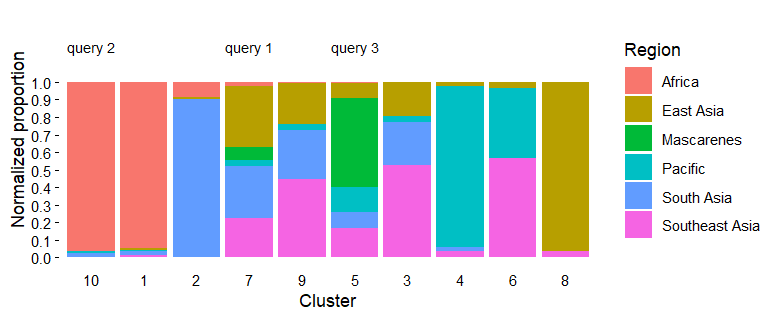

Supplement: Supplementary file 1 [file insects-16-01271-s001.zip › insects-3760316-supplementary/Supplementary Material/Supplementary Files_Vanbergen_et_al_2025_Insects/FIle_S1_DAPC_script/example_output/DAPC_assignment.png]

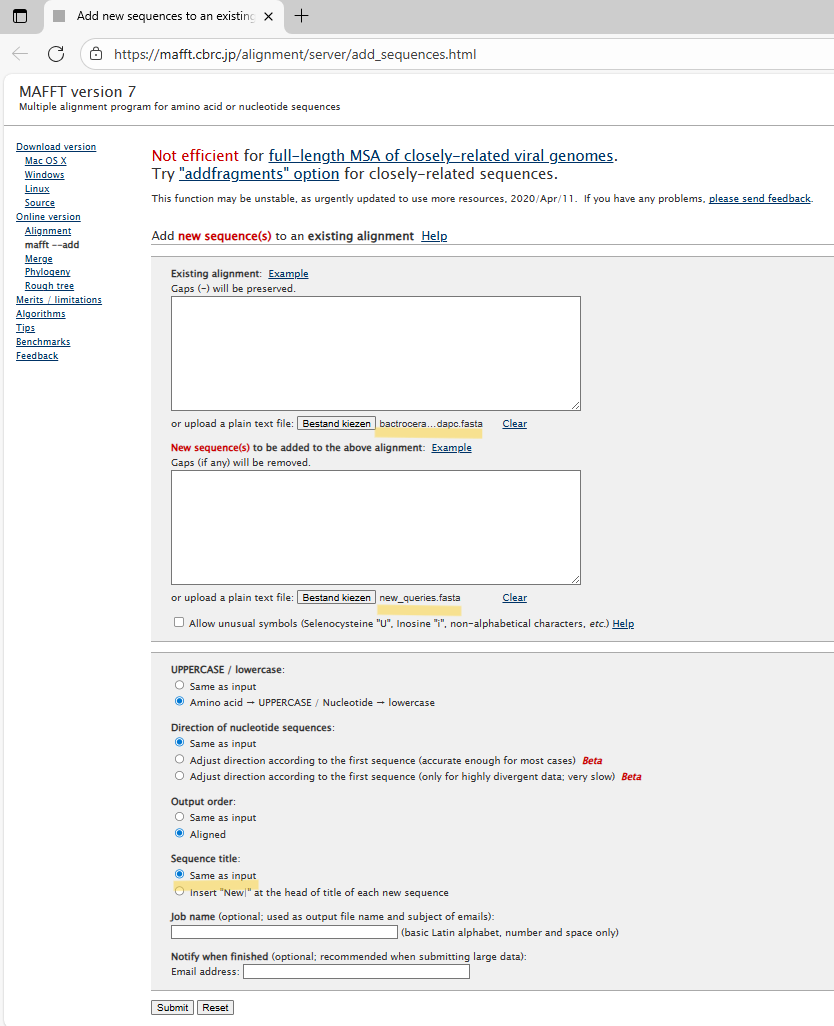

Supplement: Supplementary file 1 [file insects-16-01271-s001.zip › insects-3760316-supplementary/Supplementary Material/Supplementary Files_Vanbergen_et_al_2025_Insects/FIle_S1_DAPC_script/screenshot_MAFFT_add_sequences_part_1.png]

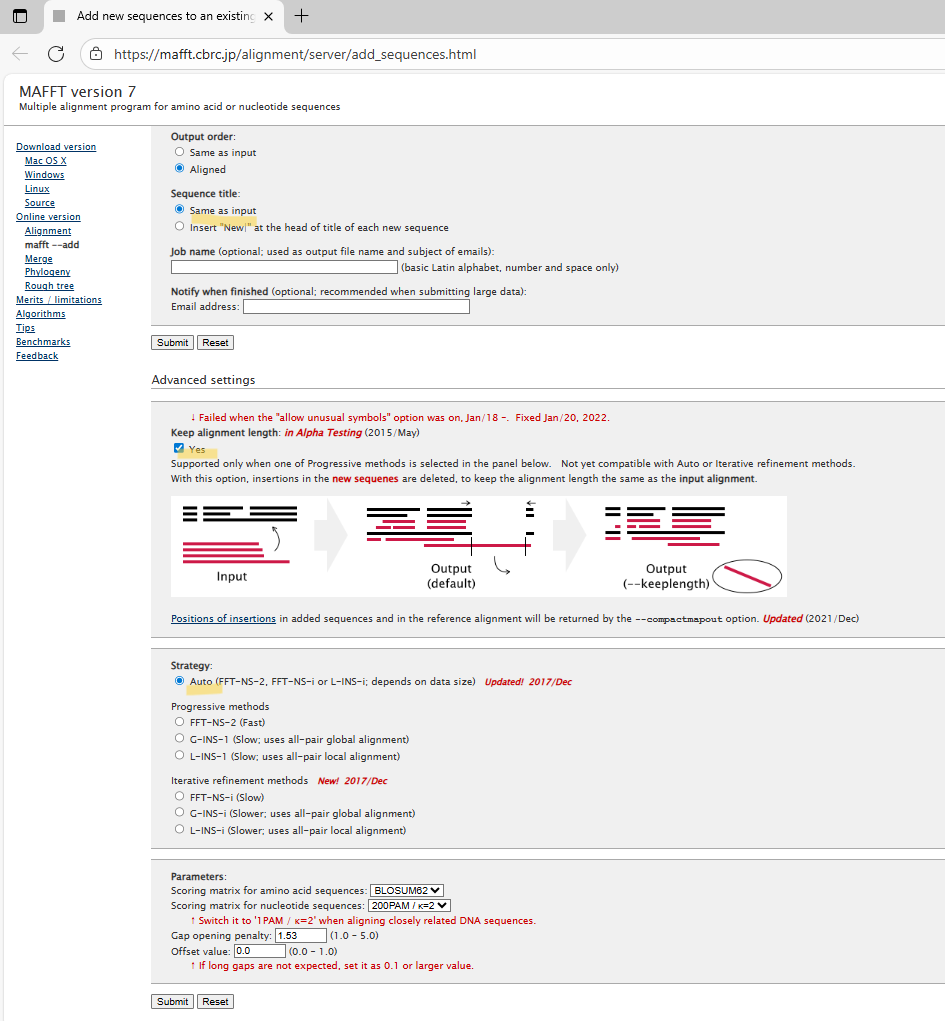

Supplement: Supplementary file 1 [file insects-16-01271-s001.zip › insects-3760316-supplementary/Supplementary Material/Supplementary Files_Vanbergen_et_al_2025_Insects/FIle_S1_DAPC_script/screenshot_MAFFT_add_sequences_part_2.png]
